# Passenger Flow Forecasting in Metro Transfer Station Based on the Combination of Singular Spectrum Analysis and AdaBoost-Weighted Extreme Learning Machine

**DOI:** 10.3390/s20123555

**Published:** 2020-06-23

**Authors:** Wei Zhou, Wei Wang, De Zhao

**Affiliations:** 1 School of Transportation, Southeast University, Nanjing 211189, China; veager@seu.edu.cn (W.Z.); zhaode@seu.edu.cn (D.Z.); 2 Jiangsu Key Laboratory of Urban ITS, Nanjing 211189, China; 3 Jiangsu Province Collaborative Innovation Centre of Modern Urban Traffic Technologies, Nanjing 211189, China

**Keywords:** automatic fare collection system, passenger flow forecasting, time series decomposition, singular spectrum analysis, ensemble learning, extreme learning machine

## Abstract

The metro system plays an important role in urban public transit, and the passenger flow forecasting is fundamental to assisting operators establishing an intelligent transport system (ITS). The forecasting results can provide necessary information for travelling decision of travelers and metro operations of managers. In order to investigate the inner characteristics of passenger flow and make a more accurate prediction with less training time, a novel model (i.e., SSA-AWELM), a combination of singular spectrum analysis (SSA) and AdaBoost-weighted extreme learning machine (AWELM), is proposed in this paper. SSA is developed to decompose the original data into three components of trend, periodicity, and residue. AWELM is developed to forecast each component desperately. The three predicted results are summed as the final outcomes. In the experiments, the dataset is collected from the automatic fare collection (AFC) system of Hangzhou metro in China. We extracted three weeks of passenger flow to carry out multistep prediction tests and a comparison analysis. The results indicate that the proposed SSA-AWELM model can reduce both predicted errors and training time. In particular, compared with the prevalent deep-learning model long short-term memory (LSTM) neural network, SSA-AWELM has reduced the testing errors by 22% and saved time by 84%, on average. It demonstrates that SSA-AWELM is a promising approach for passenger flow forecasting.

## 1. Introduction

As an import part in urban public transit, metro transit has developed rapidly and attracted a quantity of passengers in recent years. It is a great challenge for operators and design-makers to optimize the metro schedules and organize the passengers in the stations effectively. Accurate and timely short-term passenger flow forecasting is the fundament of intelligent transport systems (ITS) [1]. The prediction results not only offer evidence for passenger guidance to prevent congestion and trampling [2] but, also, provide necessary information for the metro schedule coordination scheme to match the metro capacity with the passenger flow demand.

As the connections of different metro lines, transfer stations are crucial in metro networks. Some researchers utilized the complex network theory to investigate the characteristics of the metro networks such as Beijing [3], Shanghai [4], Guangzhou [5], and some other cities [6]. The findings of their studies indicated that transfer stations played the most significant role in the networks. Some of them [3,4] suggested that the transfer stations should be paid more attention to. In addition, the passenger flow in the transfer station is usually much larger than that in a regular station, and the passenger flow increases more rapidly at the rush hours in the morning and evening. This is because transfer stations are usually located in areas with large travel demands—for instance, a transportation hub, business district, and so forth. Therefore, in order to avoid pedestrian congestion or early warnings of burst passenger flows for operators, it is vital to forecast the passenger flow accurately and timely in a transfer station.

The passenger flow is defined as the number of boarding or alighting pedestrians at the target station during a constant interval in the prediction tasks [7,8]. In previous studies, the collection of passenger flows mainly includes two ways, as follows:

1. **Videos.** The passenger flow videos are generally used to extract the passenger trajectories through image-processing techniques. The extracted data can help researchers to investigate and analyze passenger behaviors [9].
2. **Automatic Fare Collection (AFC) systems.** Based on AFC systems, the passenger boarding and alighting information is recorded by the sensors in turnstiles automatically, and the recorded data is easy to access. The AFC systems are initially designed and employed to charge the passengers automatically. Since the AFC systems can also record some extra information of the passengers (i.e., personal identification, boarding/alighting time, boarding/alighting station, etc.), the AFC data has been used in the researches of transportation engineering. These studies are mainly focused on four fields: prediction of passenger flow [2,7,10,11,12], analysis of passenger flow patterns [13], investigation of passenger behaviors [14,15], and evaluation of metro networks [3,6].

The task of passenger flow prediction is quite similar to traffic flow prediction [7,8,12,16], which is only different in the input data of the models. Therefore, many practical models of traffic flow prediction could be referred to as well. In the studies to date, the passenger/traffic flow prediction approaches are roughly classified into four categories, as listed below:

1. **Parametric models.** Due to a low computation complexity, parametric models are widely used in early studies—for instance, autoregressive integrated moving average (ARIMA) [17,18], Kalman filter (KF) [11], exponential smoothing (ES) [19], and so on. However, these models are sensitive to passenger flow patterns, since they are established based on the assumption of linearity.
2. **Nonparametric models.** In order to capture the nonlinearity of passenger flow, the nonparametric models are introduced in subsequent researches, such as *K*-nearest neighbor (KNN) [20,21], support vector regression (SVR) [7,10], artificial neural network (ANN) [1,22], etc. The empirical results from these studies have suggested that the nonparametric models usually performed better than parametric models when the data size was large. It is owing to the ability of nonlinearity modeling.
3. **Hybrid models.** The hybrid models are the combination of two or more individual methods. Due to both the linearity and nonlinearity of passenger flow, the hybrid models [2,23,24,25,26] are proposed to capture these two natures to increase the prediction accuracy. Both theoretical and empirical findings have demonstrated that the integration of different models can take full advantage of these models. Thus, this is an effective way to improve the predictive performance.
4. **Deep-learning models.** Besides the aforementioned three kinds of models, according to the latest researches, the deep-learning methods have been introduced and developed in the passenger flow forecasting problem, including long short-term memory (LSTM) [12,16,27], deep belief network (DBN) [28], stacked autoencoders (SAE) [29], convolutional neural network (CNN) [12,30], etc. Due to the universal approximation capability of complex neural networks, the deep-learning models can approximate any nonlinear function in theory [24,31]. From the findings of these studies, deep-learning models usually show a superiority of high forecasting accuracy to parametric and nonparametric models. However, because of high computation complexity, the deep-learning models will require significant resources and training time [32]. In addition, these models are usually regraded as a “black box” [23] and lack interpretability of the results [32].

In recent studies, the combination of time series decomposition approaches is a novel research interest of the hybrid models to make a better predictive performance. The principle of this kind of model is that a complicated time series can be simplified through disaggregating the sequence into multiple frequency components. The decomposed components are forecasted separately, and then, these predicted results are summed as the final outcomes. The widely used time series decomposition methods include: wavelet decomposition (WD) [25,33], empirical mode decomposition (EMD) [2,26,34], Seasonal and Trend Decomposition Using Loess (STL) [35,36], singular spectrum analysis (SSA) [37,38,39], and so on. Sun et al. [25] and Liu et al. [33] employed the WD approach to decompose the original passenger flow into several high-frequency and low-frequency sequences, and then, these sequences were forecasted based on least squares SVR by Sun et al. [25] and extreme learning machine (ELM) by Liu et al. [33], respectively. Chen et al. [2], Wei and Chen [26], and Chen and Wei [34] all proposed that the passenger flow could be regarded as a nonlinear and nonstationary signal, and they utilized EMD to decompose the original passenger flow into nine intrinsic mode functions (IMF) components and one residue. Wei and Chen [26] predicted the disaggregated components through ANN, while Chen et al. [2] predicted them through LSTM. Qin et al. [35] utilized STL to disaggregate the monthly air passenger flow into three subseries: seasonal, trend, and residual series. Then, they developed the Echo State Network (ESN) to forecast each decomposed series. Chen et al. [36] also employed STL to decompose the daily metro ridership, and LSTM was used in the prediction stage. As for the SSA method, to the best of our knowledge, this method has never been introduced to an analysis passenger flow to date, although this method was devolved for traffic flow prediction. Mao et al. [37], Shang et al. [38], and Guo et al. [39] all have developed this method to analyze the traffic flow time series and obtained several components with different amplitudes and frequencies. Then, they reconstructed these components into a smoothing part and residue. In this way, the SSA could be regarded as a filter to remove noise from the original sequence. During the stage of forecasting, the denoise data was predicted by ELM [38] and a grey system model [39], respectively. Overall, these studies have clearly indicated that the hybridization of time series decomposition approaches can make an obvious improvement on the predictive accuracy. However, all the aforementioned literatures have failed to investigate the potential characteristic of passenger flow from the decomposed results.

In this study, a novel hybrid model (i.e., SSA-AWELM), SSA combined with an AdaBoost-weighted extreme learning machine (AWELM), is proposed to achieve more accurate predicted results for the metro passenger flow. The experimental data, recorded by the sensors in turnstiles, is collected from an AFC system. The main works of this paper are briefly described as follows:

1. The SSA approach is developed to decompose the original passenger into three components: trend, periodicity, and residue. Investigation of the three components can discover the inner characteristics of the original data.
2. The ELM improved by AdaBoost (i.e., AWELM) is developed to forecast the three components. ELM, a neural network famous for fast computer speeds, is implemented, and the prediction performance is enhanced through AdaBoost ensemble learning. Thus, the hybrid model SSA-AWELM has the advantage of both accuracy and speediness for passenger flow forecasting.
3. Multistep-ahead prediction of the passenger flow is established, which can offer more information of the future. A dataset collected from a metro AFC system is utilized to carry out the prediction tests and comparative analysis.

The rest of this paper is organized as follows: In Section 2, the problem is defined, and the proposed method is formulated. In Section 3, the procedures of data collection, data preprocessing, and design of the experiment are elaborated. The results and findings are analyzed and discussed in Section 4. At last, the conclusions are drawn in Section 5.

## 2. Materials and Methods

In this section, the AFC system is briefly introduced, and the passenger flow forecasting problem is explained in detail. In particular, the model SSA-AWELM is formulated to improve upon the performance of predictions.

### 2.1. Automatic Fare Collection Systems

**Figure 1:**
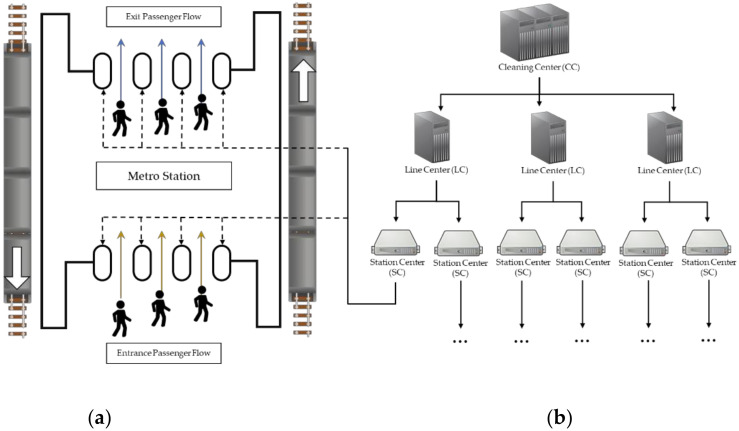
Brief structure of an automatic fare collection (AFC) system: (**a**) metro station and (**b**) computer cluster.

The automatic fare collection (AFC) systems are established on the Internet of Things (IoT) and wireless sensor networks (WSN). As displayed in Figure 1, a typical AFC system consists of five hierarchical levels: cleaning center (CC), line centers (LC), station computers (SC), station equipment, and smart tickets and cards, from top to bottom [40]. A passenger touches a smart ticket or card, which has an integrated circuit (IC) clip (a type of microsensor) inside, to a turnstile when boarding or alighting; meanwhile, the sensor in the turnstile will respond and record some necessary information. Then, the information will be transmitted to the SC, LC, and, finally, to the CC. In addition, there are a few differences between boarding and alighting. When the passenger alights and passes a turnstile, the sensor will compute the traveling mileage and charge the fare automatically, and this transaction could be completed in milliseconds.

The AFC system can not only be employed by operators to collect the fares from passengers conveniently. For researchers, what is the most important is that the data mining results from the recorded information could assist with analyzing the operational quality, since the records include the personal identification, boarding/alighting station, boarding/alighting time, and some other useful information. Based on AFC systems, the passenger boarding and alighting information could be recorded by the sensors in turnstiles automatically, and the recorded data could be accessed easily. This makes it possible to realize real-time predictions of the metro passenger flow.

### 2.2. Passenger Flow Forecasting Problem

As mentioned in Section 1, passenger flow is the sum of boarding or alighting pedestrians during a constant interval (i.e., 5 min, 10 min, etc.) in the target station. Suppose *x_t_* denotes the entrance or exit passenger flow at the time *t*, then it is obvious that *x_t_* varies with the time. The passenger flow forecasting problem can be treated as a time series forecasting task, and the passenger flow time series takes the instinct of temporal dependence. In other words, the passenger flow is highly related to the historical data. Therefore, the research problem addressed in this paper is to forecast *x_t_* by the historical passenger flow data {*x_t_*_−1_, *x_t_*_−2_, *x_t_*_−3_, …, *x_t_*_−*n*_}, which is formulated as follows:

(1) $${\hat{x}}_{t}=E\left(x_{t-1},x_{t-2},\ldots ,x_{t-n}\right)$$

where ${\hat{x}}_{t}$ represents the predictive value at time *t*, $E\left(\cdot \right)$ represents an established prediction model, and *n* represents the order of time lagging.

Although the single-step passenger flow forecasting has been widely studied, in order to provide travelers and managers with further information about passenger flow, multistep forecasting is necessary. In our study, the iterated strategy, which is widely used in time series predictions [41,42], is adopted for multistep passenger flow forecasting. As Equation (2) expresses, based on the established model with single-step prediction, the iterated strategy inputs a prediction value into the same model to forecast the value at the next time. It continues in this manner until reaching the maximum prediction horizon. The iterated strategy has two outstanding advantages. One is that the model just requires being trained once, and the other is that the prediction steps are unlimited.

(2) $${\hat{x}}_{t+1}=E\left({\hat{x}}_{t},x_{t-1},\ldots ,x_{t-n+1}\right)\;{\hat{x}}_{t+2}=E\left({\hat{x}}_{t+1},{\hat{x}}_{t},\ldots ,x_{t-n+2}\right)\;\ldots$$

### 2.3. The Proposed Hybrid Model

#### 2.3.1. Singular Spectrum Analysis

Singular spectrum analysis (SSA) is a time series analysis approach without any statistical assumptions [43]. It can decompose the original data into several components. This method has been widely used to decompose the time series including traffic flow [37,38,39]. In this study, this approach is implemented to analyze the passenger flow. Suppose ***Y***(*t*) (*t* = 1, 2, …, *N*) denotes the original passenger flow sequence with length *N*. The processes of the SSA approach contains four steps, as follows:

**Step 1: Embedding**

The original sequence ***Y***(*t*) is transformed into the trajectory matrix $F\in {\mathbb{R}}^{L\times K}$, which is calculated as the following equation:

(3) $$F=\left[\begin{array}{cccc} f_{1} & f_{2} & \cdots & f_{K}\\ f_{2} & f_{3} & \cdots & f_{K+1}\\ \vdots & \vdots & \ddots & \vdots \\ f_{L} & f_{L+1} & \cdots & f_{N} \end{array}\right]$$

where *L* is window length, *K* = *N* – *L* + 1, and *f_i_* is the *i*th (1≤i≤N) value of the original sequence.

**Step 2: Singular Value Decomposition (SVD)**

The SVD algorithm is conducted to decompose the trajectory matrix ***F***, computed as follows:

(4) $$F=U\cdot \Sigma \cdot V^{T}=\sum_{i=1}^{d}\sqrt{\lambda_{i}}U_{i}V_{i}{}^{T}$$

where ***Σ*** is diagonal matrix, and the diagonal elements ($\sqrt{\lambda_{1}}\geq \sqrt{\lambda_{2}}\geq \ldots \geq \sqrt{\lambda_{d}}\geq 0$) are the singular values of ***F***. Vectors ***U****_i_* and ***V****_i_*, which are the *i*th column of matrix ***U*** and ***V***, represent the left and right singular vectors, respectively. *d* represents the number of singular values, and it is also the rank of trajectory matrix ***F***. The collection $\left\{U_{i},\sqrt{\lambda_{i}},V_{i}\right\}$ is denoted as the *i*th eigen triple of SVD.

Every eigen triple can reconstruct an elementary matrix ***F****_i_* of trajectory matrix ***F***:

(5) $$F_{i}=\sqrt{\lambda_{i}}U_{i}V_{i}^{T}$$

Thus, the sum of all elementary matrixes ***F****_i_* is identical to the trajectory matrix ***F***. The contribution of elementary matrix ***F****_i_* is measured by the corresponding eigen value (equal to the square of the singular value) as the following equation:

(6) $$\eta_{i}=\frac{\lambda_{i}}{\sum_{i=1}^{d}\lambda_{i}}$$

**Step 3: Grouping**

Indices set *D* = {1, 2, …, *d*} is divided into *M* disjointed subsets *I*_1_, *I*_2_, …, *I_M_*. Every indices subset *I_m_* (*m* = 1, 2, …, *M*) is regarded as one group, and the elementary matrixes ***F****_i_* ($i\in I_{m}$) in each group are summed. In previous papers, the w-correlation method [43] is prevalent to split the results set. However, this method is conducted from the perspective of signal analysis, which lacks the interpretability for passenger flow. In this study, the elementary matrixes ***F****_i_* are grouped into three parts of trend ***F****_T_*, periodicity ***F****_P_*, and residue ***F****_R_*, expressed as Equation (7), and this process is detailed in Section 4.1.

(7) $$F=F_{T}+F_{P}+F_{R}$$

**Step 4: Diagonal averaging**

The grouped matrixes ***F****_i_* ($F_{i}\in \left\{F_{T},F_{P},F_{R}\right\}$) are transformed into the one-dimensional time series format by diagonal averaging. Assume *f_ij_* (1 ≤ *i* ≤ *L*, 1 ≤ *j* ≤ *K* ) is the element of matrix ***F****_i_*, $L^{*}=\min(L,K),K^{*}=\max(L,K)$, and $f_{ij}^{*}=f_{ij}$, if *K* > *L*; otherwise, $f_{ij}^{*}=f_{ji}$. Then, every element *y_i_* of the time series ***Y_i_***(*t*) is computed as the following equation:

(8) $$y_{t}=\left\{\begin{array}{ll} \frac{1}{t}\sum_{m=1}^{t}f_{m,t-m+1}^{*} & 1\leq t<L^{*}\\ \frac{1}{L^{*}}\sum_{m=1}^{L^{*}}f_{m,t-m+1}^{*} & L^{*}\leq t\leq K^{*}\\ \frac{1}{N-t+1}\sum_{m=t-K^{*}+1}^{N-K^{*}+1}f_{m,t-m+1}^{*} & K^{*}<t\leq N \end{array}\right.$$

As such, the original passenger flow ***Y***(*t*) is disaggregated into three components of trend ***T***(*t*), periodicity ***P***(*t*), and residue ***R***(*t*).

#### 2.3.2. AdaBoost Ensemble Learning

As a strategy of ensemble learning, AdaBoost was originally proposed by Freund and Schapire [44] for classification problems. Drucker [45] developed this algorithm in the application of a regression problem, and it was improved upon by Solomatine and Shrestha [46,47]. With the integration of a few homogenous models (called base learners), this method can improve the performance of base learners. In this study, the AdaBoost algorithm is utilized to assist the ELM to predict the passenger flow more accurately.

Supposing a dataset ${\left\{\left(x_{i},y_{i}\right)\right\}}_{i=1}^{N}$ with *N* samples, *T* is the maximum iteration number. The specific steps of AdaBoost is presented as the following:

**Step 1:** Initialize the distribution of sample weights:

(9) $$\Gamma_{1}={\left[\gamma_{1,1},\gamma_{1,2},\ldots ,\gamma_{1,N}\right]}^{T},\mathrm{where}\;\gamma_{1,n}=\frac{1}{N},n=1,2,\ldots ,N$$

**Step 2:** For the training process of each iteration, *t* = 1, 2, …, *T*.

**Step 2.1:** Use the dataset with a distribution of ***Γ****_t_* to train the WELM and obtain the base learner *E_t_*(***x***).

**Step 2.2:** Calculate the absolute relative error of each sample and the error rate of *E_t_*(***x***):

(10) $$\varepsilon_{t}=\overset{N}{\underset{n=1}{\sum }}\gamma_{t,n}n:\left|\frac{E_{t}\left(x_{n}\right)-y_{n}}{y_{n}}\right|>\varphi$$

where $\left|\left(E_{t}\left(x_{n}\right)-y_{n}\right)/y_{n}\right|$ represents the absolute relative error of each sample; *ε_t_* is the error rate of *E_t_*(***x***); and *n* = 1, 2, …, *N* is the index of the sample. $n:\left|\left(E_{t}\left(x_{n}\right)-y_{n}\right)/y_{n}\right|>\varphi$ represents that only the error for any particular sample is greater than the preset error, the so-called threshold *φ*; the corresponding sample will be considered. *φ* is a preset parameter and will be discussed at the end of the present subsection. More details are described in [47].

**Step 2.3:** Calculate the coefficient for updating the sample weights:

(11) $$\beta_{t}=\varepsilon_{t}^{k}$$

where *k* is the power coefficient of error rate *ε_t_* requiring to be preset. According to the study of Solomatine and Shrestha [47], *k* is selected from 1 (linear law), 2 (square law), and 3 (cubic law). A high value of *k* may cause the algorithm to become unstable. Thus, *k* is set as 1 in our study.

**Step 2.4:** Update the distribution of sample weights:

(12) $$\gamma_{t+1,n}=\frac{\gamma_{t,n}}{Z_{t}}\times \left\{\begin{array}{ll} \beta_{t}, & if\left|\frac{E_{t}\left(x_{n}\right)-y_{n}}{y_{n}}\right|\leq \varphi \\ 1, & otherwise \end{array}\right.,n=1,2,\ldots ,N$$

where *Z_t_* is a normalization factor, such that $\sum_{n=1}^{N}\gamma_{t+1,n}=1$.

**Step 3:** Update *t* = *t* + 1 and loop **Step 2.1** to **2.4** until reaching the maximum iteration number *T*. Finally, the output is computed as:

(13) $$g(x)=\frac{1}{\sum_{t=1}^{T}\ln\frac{1}{\beta_{t}}}[\sum_{t=1}^{T}(\ln\frac{1}{\beta_{t}})E_{t}(x)]$$

The AdaBoost algorithm is sensitive to the threshold *φ*. If the *φ* is too low, the model will be underfitting. On the other hand, too high a value of *φ* will raise overfitting problems. In our study, the threshold *φ* is set adaptively according to the median of absolute relative errors *ε_t_* during each iteration, expressed as the following equation:

(14) $$\varphi =\mathrm{median}\left\{\varepsilon_{1},\varepsilon_{2},\ldots ,\varepsilon_{N}\right\}$$

As presented in the above steps, AdaBoost is an iteration process. The base learner will be trained, and the distribution of the sample weights will be updated during each iteration. Thus, if the base learner is complex and spends lots of computing time, the consuming time of AdaBoost will increase linearly. In this study, ELM is adopted as the base learner, which is famous for its fast training speed. This model is elaborated in the next subsection.

#### 2.3.3. Weighted Extreme Learning Machine

Extreme learning machine (ELM) is a kind of single hidden layer feed-forward network (SLFN), which is proposed by Huang at el. [48]. Compared with traditional ANN models, ELM does not need to tune the input weights and hidden layer biases during training. After the initialization of the ELM, the input weights and hidden biases are fixed, and only the output weights are optimized. Therefore, the training process of ELM is faster than the traditional ANN model. Since weighted samples are used to train the base learners of AdaBoost, the weighted extreme learning machine (WELM) is developed in this study.

Assuming a weighted dataset ${\left\{\left(x_{i},y_{i},\gamma_{i}\right)\right\}}_{i=1}^{N}$ with *N* samples, and $x_{i}={\left[x_{i,1},x_{i,2},\ldots ,x_{i,P}\right]}^{T}\in \;{\mathbb{R}}^{P\times 1}$ and $y_{i}={\left[y_{i,1},y_{i,2},\ldots ,y_{i,Q}\right]}^{T}\in \;{\mathbb{R}}^{Q\times 1}$, *γ_i_* represent the input vector, output vector, and sample weights, respectively. The output of ELM with *h* hidden neurons is expressed as:

(15) $$f\left(x_{i}\right)=\overset{H}{\underset{h=1}{\sum }}\beta_{h}g\left(w_{h}x_{i}+b_{h}\right),i=1,2,\ldots N$$

where $w_{h}={\left[w_{h,1},w_{h,2},\ldots ,w_{h,3}\right]}^{T}$ represents the connection weights from the input layer to the *h*th hidden neuron; *b_h_* represents the bias in the *h*th hidden neuron; $\beta_{h}={\left[\beta_{h,1},\beta_{h,2},\ldots ,\beta_{h,Q}\right]}^{T}$ represents the connection weights from the *h*th hidden neuron to the output layer; and *g*(·) is the activation function, and the *sigmoid* function is adopted in this study, which is formulated as $g\left(\cdot \right)=1/\left(1+e^{-x}\right)$. Since ***w****_h_* and ***b****_h_* are assigned initially, Equation (15) can be simplified as:

(16) Hβ=Y

where $\beta ={\left[\beta_{1},\beta_{2},\ldots ,\beta_{H}\right]}^{T}$; $Y={\left[y_{1},y_{2},\ldots ,y_{N}\right]}^{T}$; and ***H*** is the output matrix of the hidden layer, expressed as:

(17) $$H=\left[\begin{array}{ccc} g\left(w_{1}x_{1}+b_{1}\right) & \cdots & g\left(w_{H}x_{1}+b_{H}\right)\\ \vdots & \ddots & \vdots \\ g\left(w_{1}x_{N}+b_{1}\right) & \cdots & g\left(w_{H}x_{N}+b_{H}\right) \end{array}\right]$$

The purpose of ELM is to optimize ***β*** with the object of the minimum mean square error cost function, which is expressed as $\underset{\beta }{\min}\|H\beta -Y^{2}\|$. Furthermore, when the samples are weighted with ***Γ***, the loss function of every sample requires multiplying with the corresponding sample weight, formulated as:

(18) $$\underset{\beta }{\min}\mathrm{diag}\left(\Gamma \right)H\beta -Y^{2}$$

where diag(***Γ***) is the diagonal matrix with the diagonal of ***Γ***, and the solution of Equation (18) is:

(19) $$\beta ={\left(H^{T}\mathrm{diag}\left(\Gamma \right)H\right)}^{-1}H^{T}\mathrm{diag}\left(\Gamma \right)Y$$

Overall, the output weights ***β*** of the WELM can be computed according to Equation (19) directly. It is different to the training process of traditional ANN, which is an iteration process to update connecting weights and neuron biases. This is the reason why ELM costs much less computing time than the traditional ANN.

#### 2.3.4. The Hybrid Model

**Figure 2:**
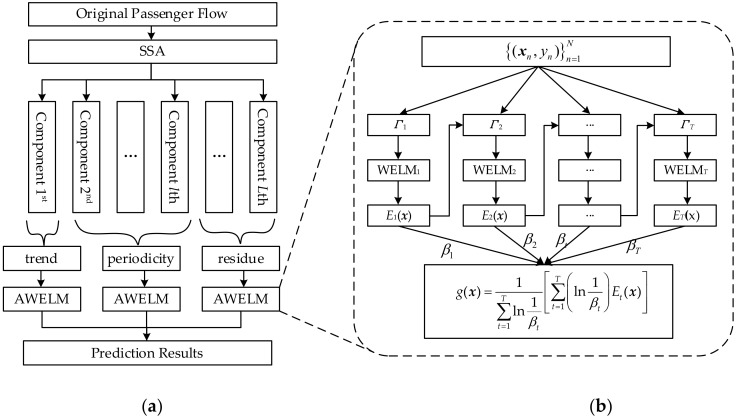
The flow chart of the hybrid singular spectrum analysis-AdaBoost-weighted extreme learning machine (SSA-AWELM) model: (**a**) SSA-AWELM and (**b**) AWELM.

The model combination of a singular spectrum analysis and AdaBoost-weighted extreme learning machine is proposed to forecast the passenger flow in this paper, symbolized as SSA-AWELM. The flow chart of this hybrid model is displayed in Figure 2, and the special process of it is described as follows:

**Step 1: SSA for decomposition.** The original passenger flow is decomposed into several components by SSA approach, and these components are grouped into three parts of trend, periodicity, and residue.

**Step 2: AWELM for components forecasting.** The WELM improved by AdaBoost (AWELM) is implemented to model and predict the three components, separately.

**Step 3: Integration for final forecasting results.** The final outcomes of forecasting the passenger flow are calculated by summing the predicted results of the three components.

## 3. Empirical Study

### 3.1. Data Collection

In this paper, the passengers’ alighting and boarding dataset is collected from the AFC system of Hangzhou metro in China. The dataset is online and provided by Ali Tianchi [49]. This dataset recorded detailed information when the passengers passed the turnstiles. The duration of the data was from the 1st to 26th in January 2019. The dataset includes seven fields, and they are listed in Table 1. In addition, some samples of the dataset are provided in Table 2.

### 3.2. Data Preprocessing

**Figure 3:**
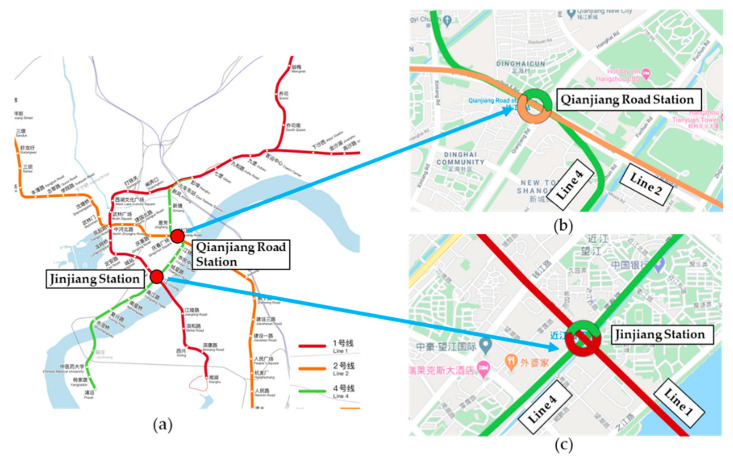
The location of the study metro transfer stations: (**a**) the Hangzhou metro network, (**b**) the Qiangjiang Road Station (Q.R. Sta.), and (**c**) the Jinjiang Road Station (J. Sta.).

The preprocessing is to obtain a passenger flow time series data from the raw AFC dataset. In this study, the passenger flow data of the Qianjiang Road Station (Q.R. Sta.) and JinJiang Station (J. Sta.) are selected to conduct experiments. As displayed in Figure 3, the Q.R. Sta. is a transfer station between Line 2 and Line 4, and it is located in the Qianjiang New Town Central Business District (CBD). The Jinjiang Station is a transfer station between Line 1 and Line 4, which is located in Wangjiang New Town.

According to previous studies [11,50], the raw recorded data are usually aggregated into 5-min intervals to obtain the passenger flow sequence. In order to keep the complete cycle periods of the sequence data, three continuous weeks, which were from the 6th to 26th of January, were selected from the AFC dataset. The time range selected was from 6:00 to 23:00 according to the operation time of the Hangzhou metro system, though a few records in the AFC dataset were out of this range. At last, there were 204 samples on average in one day and 4284 samples in total. Furthermore, the exit and entrance passenger flow sequences were computed separately. Hence, four experimental datasets were established, and they were used to test the proposed model, respectively.

**Figure 4:**
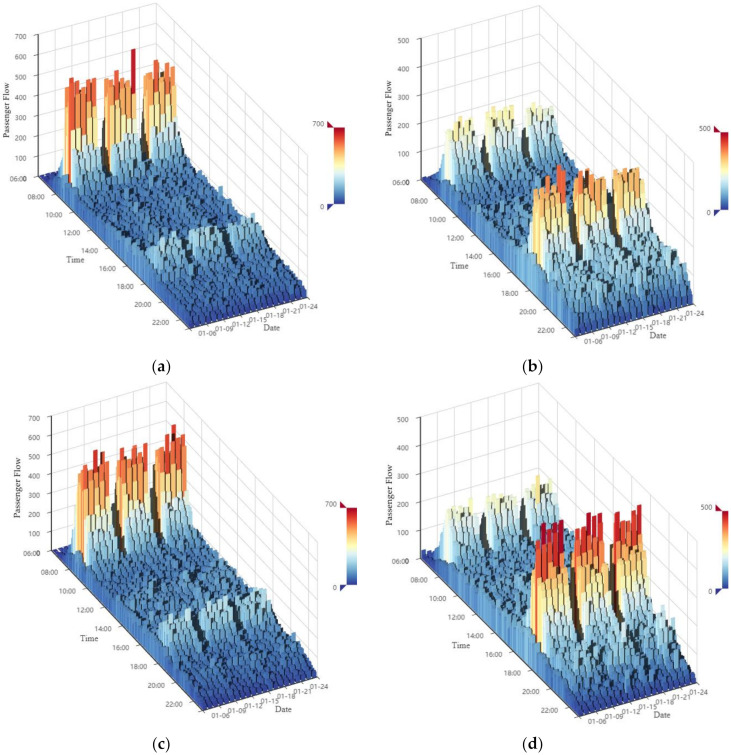
Passenger flow of the study metro transfer stations: (**a**) exit passenger flow of the Q.R. Sta., (**b**) entrance passenger flow of the Q.R. Sta., (**c**) exit passenger flow of the J. Sta, and (**d**) entrance passenger flow of the J. Sta.

The extracted passenger flow sequences are presented in Figure 4. Both the exit and entrance passenger flows on weekdays have distinct peaks in the morning (about from 8:00 to 9:00) and evening (about from 18:00 to 19:00) rush hours, while these patterns disappear on the weekends. Moreover, the peak patterns of exit and entrance passenger flows on weekdays are different. Taking the Q.R. Sta. as an example, the exit passenger flow in the morning rush hour (about 500 pedestrians per 5 min) is approximately 2.5 times larger than that in the evening rush hour (about 200 pedestrians per 5 min). On the contrary, the entrance passenger flow in the evening rush hour (about 300 pedestrians per 5 min) is approximately 1.5 times larger than that in the morning rush hour (about 200 pedestrians per 5 min). These results indicate that most passengers in this station are commuters. This finding agrees with the location of this station, i.e., it is in the Qianjiang New Town CBD and surrounded by numerous office buildings.

The four datasets are all split into training datasets (i.e., the 6th to 19th of January) and testing datasets (i.e., the 20th to 26th of January). The grid search and 5-fold cross-validation methods are used to evaluate the training performance and determine the hyper-parameters of the models. Then, the models with determined hyper-parameters are evaluated by testing the datasets.

### 3.3. Comparison Models and Evaluation Measures

In order to demonstrate the contributions of the proposed SSA-AWELM model, the classical time series model ARIMA and four extra models based on the neural network, including ANN, LSTM, ELM, and AWELM, are tested as benchmarks. They are listed as follows:

- **ARIMA:** ARIMA is a classical statistical model for time series forecasting. It is widely used to predict traffic flow and passenger flow in early studies [17]. The performance of ARIMA is affected by three parameters: autoregressive order *p*, difference order *d,* and moving average order *q*. Generally, *d* is set based on the stationarity test, and the *p* and *q* are selected from the range of [0,12] based on the Bayesian information criterion (BIC) [51].
- **ANN:** Due to the ability of nonlinearity, the ANN model is widely used in time series modeling, including passenger flow forecasting. A typical ANN model consists of three parts: one input layer, one hidden layer, and one output layer and optimized through a back-propagation algorithm (thus, it also aliased as BPNN). In this study, the ANN model is optimized by a stochastic Adam algorithm with a mean square error (MSE) loss of function. The learning rate is set as 0.001, the batch-size is 256, and the epochs is 1000.
- **LSTM:** As a prevalent deep-learning model for time series modeling, the well-designed LSTM units replace traditional neurons in a hidden layer, which can assist the LSTM model to capture the temporal characteristics. This model has also been developed to predict the passenger flow. The parameters are set identically to the ANN model.
- **ELM:** The ELM model has been elaborated in Section 2.3.3.; the individual ELM model is utilized to forecast the passenger flow as a comparison.
- **AWELM:** The AWELM is combined by AdaBoost and WELM, which has been represented in Section 2.3.3. and Section 2.3.4.

To make sure that every model can achieve the best performance, the well-established grid search and 5-fold cross-validation methods are adopted to determine the hyper-parameters. The neuron number of the hidden layers in four neural network models are all selected from 2 to 50 with step 2, and the base learner of AWELM is selected from 1 to 20 with step 1. The determined hyper-parameters of each model are displayed in the Appendix A (see Table A1). In addition, the input and the output of the models are respectively set as 12 and 1 during training, and the horizon of the multistep-ahead prediction is set as 6. In other words, the passenger flow data at the last hour is used to forecast the next half-hour.

In order to accelerate learning and convergence during the training model, the min-max normalization approach (expressed as Equation (21)) is employed to scale the input data into the range of [0,1] before feeding it into the models. In addition, to obtain the final prediction results, the outputs of the models are rescaled by the reversed min-max normalization approach (expressed as Equation (21)).

(20) $$x^{\prime }=\frac{x-\min(x)}{\max(x)-\min(x)}$$

(21) $$x=x^{\prime }\times \left(\max(x)-\min(x)\right)+\min(x)$$

In order to evaluate the performances among models, two common measures are introduced in this study. They are the mean absolute error (MAE) and root mean square error (RMSE), computed as follows:

(22) $$\mathrm{MAE}=\frac{1}{N}\overset{N}{\underset{n=1}{\sum }}\left|y_{n}-{\hat{y}}_{n}\right|$$

(23) $$\mathrm{RMSE}=\sqrt{\frac{1}{N}\overset{N}{\underset{n=1}{\sum }}{\left(y_{n}-{\hat{y}}_{n}\right)}^{2}}$$

where $y_{n}$ and ${\hat{y}}_{n}$ are the true value and predicted value, respectively, and *N* is the number of samples.

Besides the aforementioned two measures, the Diebold–Mariano (DM) test [52] is implemented to test the statistical significance between the proposed model and the benchmark models. The null hypothesis is that the prediction accuracy of the tested model *E_T_*(***x***) is equal to the reference model *E_R_*(***x***). In this study, the square error is adopted to measure the model loss, expressed as $e_{i}={\left({\hat{y}}_{i}-y_{i}\right)}^{2}$. Then, the DM statistic is defined as follows:

(24) $$\mathrm{DM}=\frac{\overset{¯}{g}}{\sqrt{{\hat{V}}_{g}/N}}$$

where $\overset{¯}{g}=\sum_{n=1}^{N}g_{n}/N$, $g_{n}={\left({\hat{y}}_{n}^{T}-y_{n}\right)}^{2}-{\left({\hat{y}}_{n}^{R}-y_{n}\right)}^{2}$, ${\hat{V}}_{g}=\gamma_{0}+2\sum_{k=1}^{P-1}\gamma_{k}$, and $\gamma_{k}$ is the autocovariance at lag *k,* expressed as $\gamma_{k}=(1/N)\sum_{i=k+1}^{N}\left(g_{i}-\overset{¯}{g}\right)\left(g_{i-k}-\overset{¯}{g}\right)$. ${\hat{y}}_{n}^{T}$ and ${\hat{y}}_{n}^{R}$ respectively represent the predicted values of model *E_T_*(***x***) and *E_R_*(***x***), *P* is the prediction horizon, and *N* is the scale of the testing data.

## 4. Results Analysis

### 4.1. Analysis of SSA Decomposition

**Figure 5:**
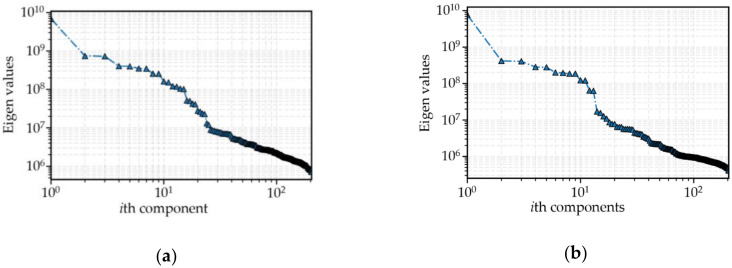
Eigen values of decomposed components (Q.R. Sta.): (**a**) exit passenger flow and (**b**) entrance passenger flow.

As mentioned in Section 2.3.1., window length *L* is the only parameter that requires to be determined before decomposition. From previous studies [37,38,39], if the time series data shows obvious periodicity, the window length *L* could be set as one period length. Thus, *L* = 204, because the passenger flow cycles daily (see Figure 4), and 204 samples on average are collected in one day (has been illuminated in Section 3.2.). Then, the original passenger flow can be disaggregated into 204 components. These components are grouped into the three parts of trend, periodicity, and residue, and this is inspired by the study [53] about using SSA to analyze the variants of electricity prices. To facilitate the analysis, taking the dataset of the Q.R. Sta. as an example, the eigen values of each component are plotted in Figure 5.

**Figure 6:**
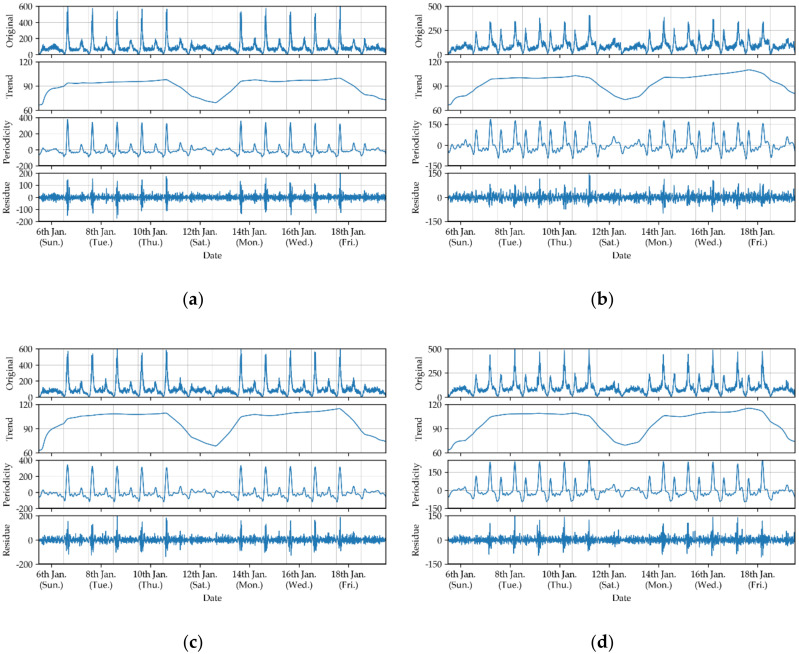
Decomposition results: (**a**) exit passenger flow of the Qianjiang Road Station, (**b**) entrance passenger flow of the Q.R. Sta., (**c**) exit passenger flow of the J. Sta., and (**d**) entrance passenger flow of the J. Sta.

Taking Figure 5a as an example, it is clear that the first eigen value is significantly larger than the others, and the corresponding component is extracted separately as trend parts. Moreover, the eigen values curve declines slowly after the 23rd component, and the 23rd is regarded as the “break point”. Then, the components from the 2nd to 23rd are reconstructed into periodic parts, and the remainder components from the 24th to 204th are reconstructed into residual parts. This is the same way as the entrance passenger flow in Figure 5b, and the “break point” is 13. Then, the components from the 2nd to 13th are reconstructed into periodic parts, and the remainder components from the 14th to 204th are reconstructed into residual parts. Finally, the obtained trend, periodicity, and residues of the original passenger flow are displayed in Figure 6.

As shown in Figure 6, every component can reveal different patterns of the original passenger flow. The trend represents the overall tendency, and the periodicity represents the variants within a day. Furthermore, it could be found in the trend that the passenger flow on weekdays is larger than that on weekends. In the periodical component, the passenger flow shows distinct peaks in the morning and evening rush hour on weekdays, but this is not obvious on the weekends. The peak patterns are different between the exit and entrance passenger flows: the exit passenger flow in the morning rush hour is much larger than that in the evening rush hour, and the entrance passenger flow is contrary to that. As for the residue, it fluctuates irregularly and can be treated as noise.

### 4.2. Analysis of Hyper-Parameters

**Figure 7:**
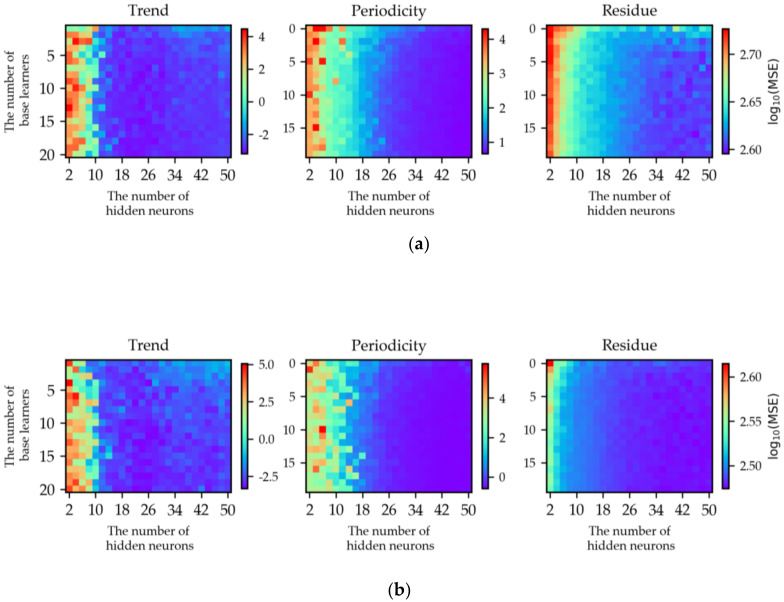
The process of hyper-parameter selection for SSA-AWELM (Q.R. Sta.): (**a**) exit passenger flow and (**b**) entrance passenger flow.

The performance of SSA-AWELM is highly dependent on the forecasting model AWELM of each component, and the AWELM has two hyper-parameters: the number of base learners (i.e., WELM) *T* and the hidden neurons of WELM *H*. The well-established grid search and five-fold cross-validation methods are adopted to determine the *T* and *H*. The *H* is selected from 2 to 50 with step 2, and *T* is selected from 1 to 20 with step 1. Taking the dataset of the Q.R. Sta. as an example, the process of hyper-parameter selection is displayed in Figure 7, and the log transformation is applied to the MSE to distinguish different values clearly. It could be found that AWELM is sensitive to the hidden neurons *H* but insensitive to base learner number *T*. Finally, the determined hyper-parameters *H* and *T* of AWELM are provided in Table A1 (see Appendix A).

### 4.3. Analysis of Forecasting Results

**Figure 8:**
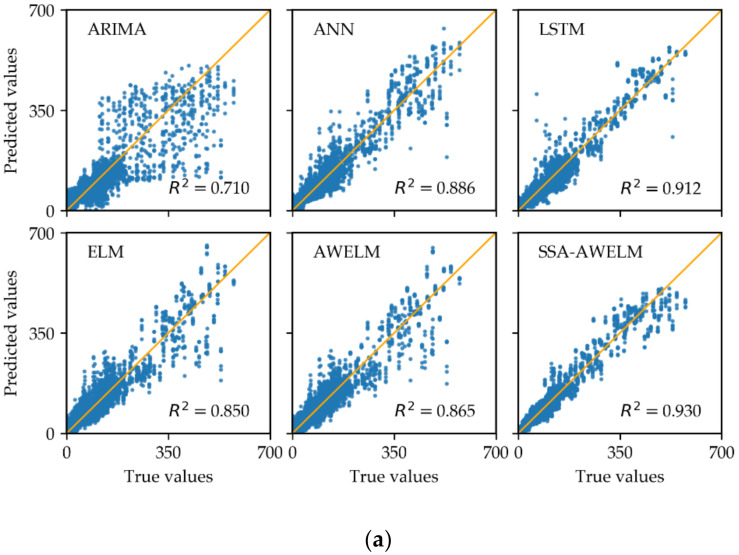

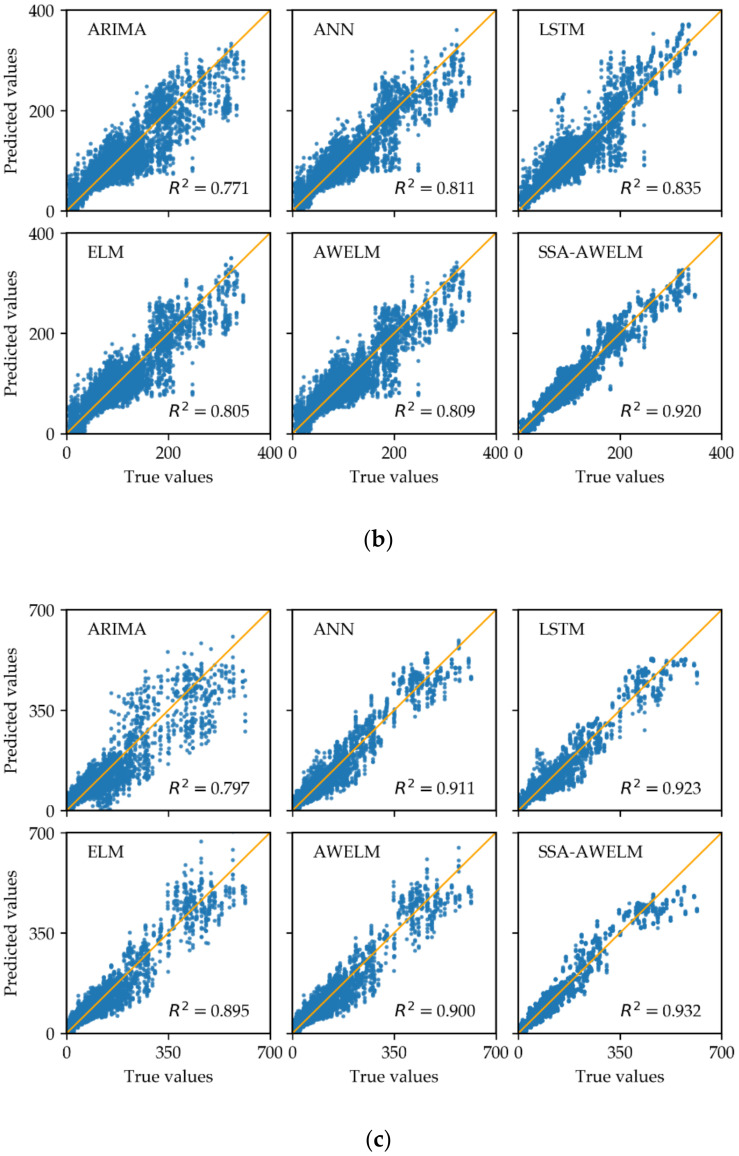

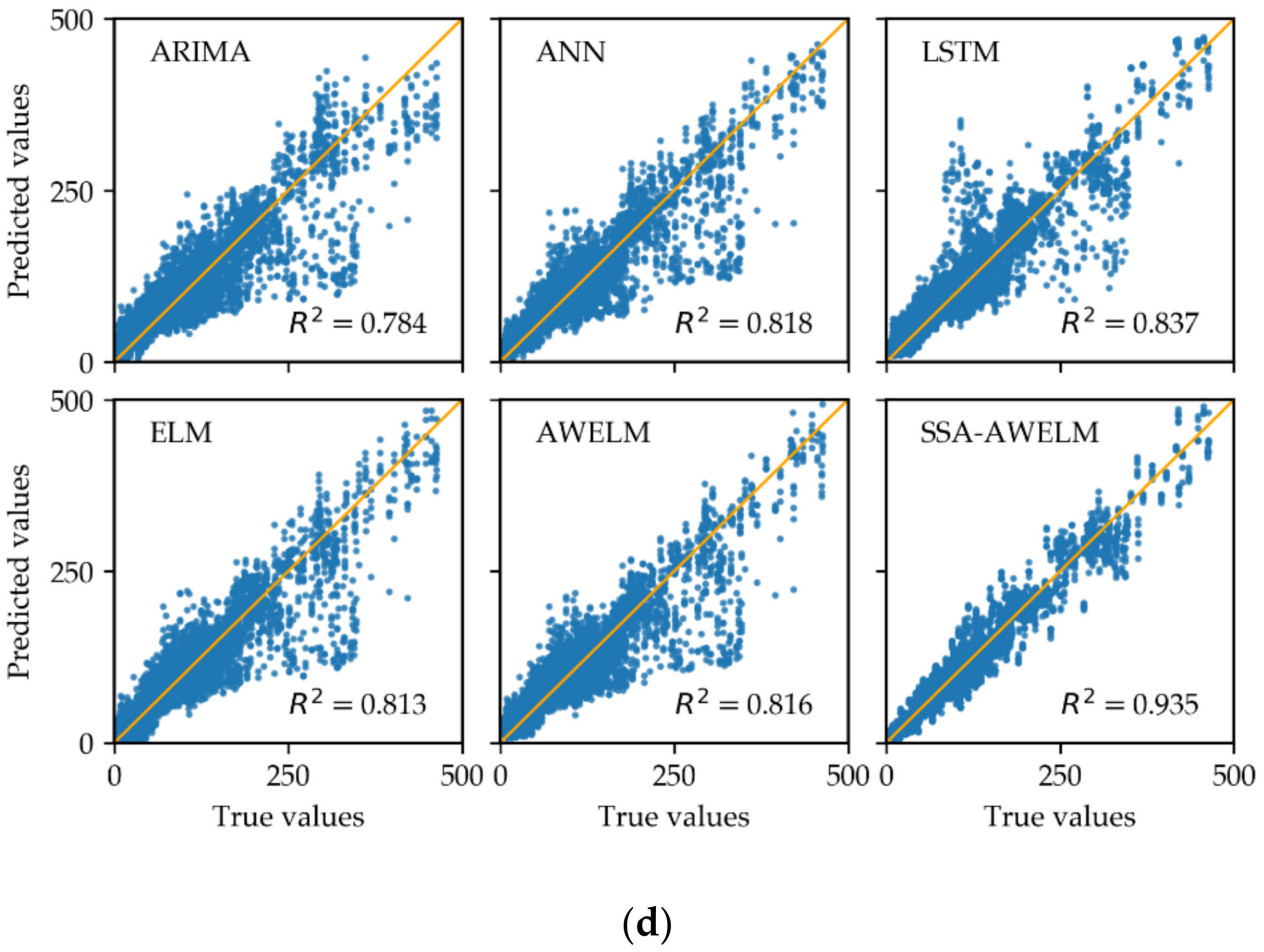
Prediction results: (**a**) exit passenger flow of the Q. R. Sta., (**b**) entrance passenger flow of the Q.R. Sta., (**c**) exit passenger flow of the J. Sta., and (**d**) entrance passenger flow of the J. Sta. ARIMA: Auto Regressive Integrated Moving Average. ANN: Artificial Neural Network. LSTM: Long Short-Term Memory neural network. ELM: Extreme Learning Machine. AWELM: AdaBoost-Weighted Extreme Learning Machine. SSA-AWELM: the proposed model combining Singular Spectrum Analysis and AdaBoost-Weighted Extreme Learning Machine.

For the sake of a comparison analysis, the average evaluation measures of the forecasting results across all the six prediction horizons are presented in Table 3, and the scatter points of the true and predicted values are displayed in Figure 8. From Table 3, it is worth noting that the proposed SSA-AWELM performs best among all the models, followed by LSTM, ANN, AWELM, ELM, and ARIMA. Compared to LSTM, the RMSE and MAE of SSA-AWELM respectively reduced by 22.5% and 21.3% on average in the case of the Q.R. Sta. and reduced by 23.6% and 20.0% on average in the case of the J. Sta. AWELM performs a little better than ELM, which indicates the AdaBoost algorithm can reduce the prediction errors but with limitations. As expected, ARIMA is always inferior to other models, because it is a linear model. In addition, it can be seen in Figure 8 that the scatter points in SSA-AWELM are closest to the expectation line, and the corresponding coefficient of determination *R*^2^ is largest. All the above findings can prove that the proposed SSA-AWELM is an effective approach to improve the accuracy of passenger flow forecasting. Furthermore, to compare the consuming time of different models, the training time is provided in Table A2 (See Appendix A).

### 4.4. Analysis of Multistep-Ahead Forecasting

**Figure 9:**
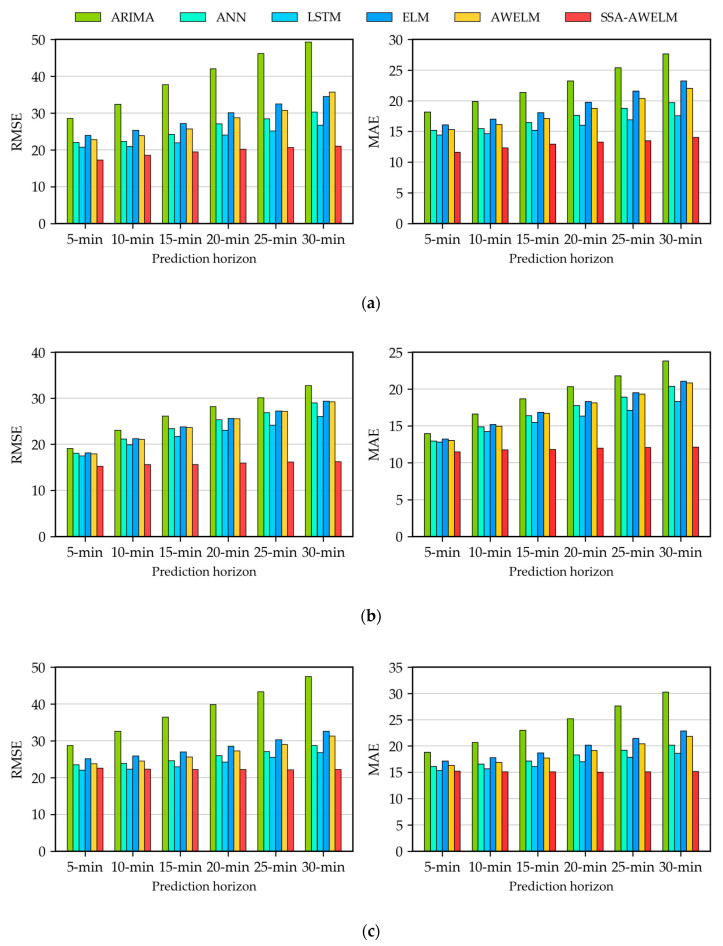

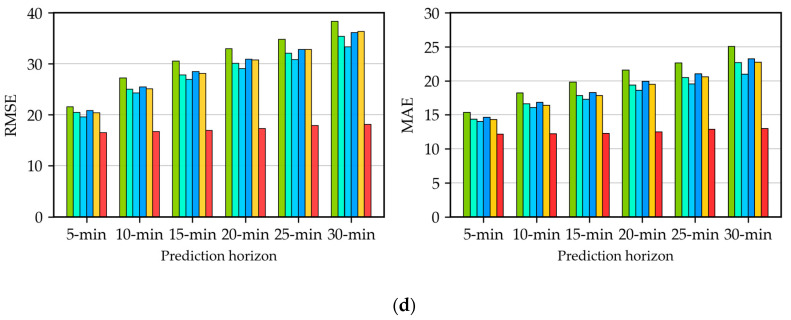
Evaluation of the multistep predictions: (**a**) exit passenger flow of the Q.R. Sta., (**b**) entrance passenger flow of the Q.R. Sta., (**c**) exit passenger flow of the J. Sta., and (**d**) entrance passenger flow of the J. Sta.

In order to analyze the multistep forecasting errors, the evaluation measures of each prediction horizon are displayed in Figure 9. The DM test results of the comparison between the proposed SSA-AWELM and benchmarks are presented in Table 4. From Figure 9, it can be seen that the prediction errors of every model increase along the prediction horizons. This is caused by the cumulative errors, which stems from feeding prediction values into the models for multistep-ahead forecasting, and the cumulative errors are inevitable. What stands out in Figure 9 is that the proposed SSA-AWELM always performs best in every prediction horizon, and the errors increase slowest in comparison with the other models. This indicates that the SSA-AWELM can improve the robustness and restrict the propagation of the cumulative error during multistep-ahead forecasting. A reasonable explanation for this finding is that the SSA can decompose the original passenger flow into the three components of trend, periodicity, and residue. Each component holds individual characteristics that can be modeled more easily than the original complex data. Furthermore, compared with ELM, AWELM preforms slightly better. It suggests that AdaBoost can improve the accuracy of ELM but with limitations. Only combining with AdaBoost cannot promote the forecasting accuracy significantly. From Table 4, generally speaking, the proposed SSA-AWELM almost always outperforms the other models at a highly significant level. There are some exceptions when compared with LSTM for the exit passenger flow. In these situations, SSA-AWELM still performs better than LSTM but not always with a highly significant level. This might because LSTM has the advantage of capturing more temporal characteristics in terms of the exit passenger flow. Overall, these findings suggest that the proposed SSA-AWELM is outstanding during multistep-ahead predictions. These results prove that the SSA-AWELM is a robust approach for passenger flow forecasting.

## 5. Conclusions

This paper studied the passenger flow forecasting and proposed a novel model SSA-AWELM. In the model, the SSA was developed to decompose the original data into the three components of trend, periodicity, and residue; then, the AWELM was developed to forecast each component separately. The three predicted results were summed as the final outcomes. In order to demonstrate the effectiveness of the proposed model, the passenger flow in two transfer stations, which were extracted from an AFC system, were utilized to carry out prediction testing and a comparison analysis. The main conclusions are drawn and listed as follows:

1. The SSA approach can get an insight into the inner characteristics of the passenger flow. The trend represents the overall tendency, the periodicity represents the variants within a day, and the residue represents noise.
2. The AWELM model, which is combined by AdaBoost and WELM, are developed to make a more accurate and faster prediction for each component. Compared to the state-of-the-art model LSTM, the propose model has improved upon the performance by 22% and saved time by 84%, on average.
3. From the results of the evaluation measures and DM statistical test, the proposed model SSA-AWELM can reduce the cumulative errors during the multistep-ahead prediction. These findings have demonstrated that the SSA-AWELM is a robust model for passenger flow forecasting.

The proposed method in this paper still retains two limitations that will be addressed in the future. One is that the testing cases are in only two transfer stations with large travel demands, and the other is that the passenger flows are collected under regular conditions. Thus, in further studies, more cases including regular stations will be tested and discussed. In addition, the passenger flows during some special incidents, such as extreme weather, passenger control, etc., will be focused on to extend the proposed model.

## Figures and Tables

**Table 1:** Data fields collected from the automatic fare collection (AFC) system of Hangzhou metro.

	Field	Description
1	Time	Passenger boarding or alighting time
2	Line ID	Number assigned to every metro line
3	Station ID	Number assigned to every metro station
4	Device ID	Number assigned to every turnstile
5	Status	Boarding or alighting: 0 represents alighting, and 1 represents boarding
6	User ID	Personal identification information
7	Pay Type	Ticket type

**Table 2:** Some samples of the collected data.

	Time	Line ID	Station ID	Device ID	Status	User ID	Pay Type
1	2019-01-01 06:00:00	B	15	759	1	Baecf***	1
2	2019-01-01 06:00:00	B	32	1558	1	Da226***	3
3	2019-01-01 06:00:01	B	8	402	1	Bb8e6***	1
4	2019-01-01 06:00:02	B	32	1562	1	C03b9***	2
5	2019-01-01 06:00:02	B	9	446	0	Be9c9***	1

**Table 3:** Average evaluation measures across all six prediction horizons. RMSE: root mean square error and MAE: mean absolute error. Q. R. Sta.: Qiangjiang Road Station and J. Sta.: Jinjiang Road Station. ARIMA: Auto Regressive Integrated Moving Average. ANN: Artificial Neural Network. LSTM: Long Short-Term Memory neural network. ELM: Extreme Learning Machine. AWELM: AdaBoost-Weighted Extreme Learning Machine. SSA-AWELM: the proposed model combining Singular Spectrum Analysis with AdaBoost-Weighted Extreme Learning Machine.

Model	Exit Passenger Flow of Q.R. Sta.		Entrance Passenger Flow of Q.R. Sta.		Exit Passenger Flow of J. Sta.		Entrance Passenger Flow of J. Sta.	
	RMSE	MAE	RMSE	MAE	RMSE	MAE	RMSE	MAE
ARIMA	40.01	22.60	26.90	19.19	38.55	24.23	31.37	20.44
ANN	25.88	17.18	24.21	16.86	25.67	17.92	28.87	18.56
LSTM	23.34	15.78	22.24	15.69	24.01	16.76	27.71	17.74
ELM	29.14	19.28	24.49	17.35	28.32	19.67	29.52	18.98
AWELM	28.22	18.28	24.35	17.15	27.00	18.71	29.37	18.55
SSA-AWELM	19.53	12.93	15.77	11.84	22.24	15.10	17.25	12.49

**Table 4:** Diebold–Mariano (DM) test results of the comparison between the proposed SSA-AWELM and benchmarks.

**Prediction** **Horizon**	**Case: Exit Passenger Flow of Q.R. Sta.**				
	**ARIMA**	**ANN**	**LSTM**	**ELM**	**AWELM**
5-min	−6.90 ***	−5.32 ***	−2.51 **	−5.54 ***	−5.07 ***
10-min	−5.58 ***	−3.75 ***	−1.88 *	−4.95 ***	−4.77 ***
15-min	−4.72 ***	−5.00 ***	−1.24	−4.98 ***	−4.91 ***
20-min	−4.13 ***	−3.29 ***	−1.77 *	−4.45 ***	−3.84 ***
25-min	−3.95 ***	−3.36 ***	−1.45	−5.06 ***	−4.39 ***
30-min	−3.78 ***	−3.00 ***	−1.42	−4.98 ***	−4.33 ***
	**Case: Entrance Passenger Flow of Q.R. Sta.**				
	**ARIMA**	**ANN**	**LSTM**	**ELM**	**AWELM**
5-min	−7.60 ***	−5.90 ***	−6.25 ***	−6.22 ***	−5.95 ***
10-min	−6.62 ***	−5.34 ***	−5.00 ***	−5.59 ***	−5.49 ***
15-min	−6.90 ***	−5.71 ***	−5.14 ***	−6.07 ***	−6.06 ***
20-min	−7.23 ***	−6.20 ***	−5.42 ***	−6.75 ***	−6.68 ***
25-min	−7.42 ***	−6.52 ***	−5.75 ***	−7.23 ***	−7.07 ***
30-min	−7.15 ***	−6.37 ***	−6.06 ***	−6.96 ***	−6.78 ***
	**Case: Exit Passenger Flow of J. Sta.**				
	**ARIMA**	**ANN**	**LSTM**	**ELM**	**AWELM**
5-min	−6.07 ***	−1.18	0.99	−2.29 **	−1.57
10-min	−5.80 ***	−2.31 **	0.15	−3.45 ***	−2.71 ***
15-min	−5.82 ***	−3.24 ***	−0.62	−4.45 ***	−3.95 ***
20-min	−5.52 ***	−4.13 ***	−1.59	−4.94 ***	−4.80 ***
25-min	−5.13 ***	−4.24 ***	−2.51 **	−4.69 ***	−5.18 ***
30-min	−4.68 ***	−4.61 ***	−2.72 ***	−4.89 ***	−5.11 ***
	**Case: Entrance passenger flow of J. Sta.**				
	**ARIMA**	**ANN**	**LSTM**	**ELM**	**AWELM**
5-min	−6.16 ***	−5.02 ***	−4.66 ***	−5.63 ***	−5.15 ***
10-min	−4.87 ***	−3.96 ***	−3.75 ***	−4.02 ***	−3.84 ***
15-min	−4.64 ***	−3.82 ***	−3.67 ***	−3.88 ***	−3.74 ***
20-min	−4.67 ***	−3.96 ***	−3.66 ***	−4.01 ***	−3.93 ***
25-min	−4.54 ***	−4.14 ***	−3.48 ***	−4.07 ***	−4.01 ***
30-min	−4.51 ***	−4.25 ***	−3.60 ***	−4.18 ***	−4.13 ***

*** represents the rejection of the null hypothesis at the 0.01 level, ** represents the rejection of the null hypothesis at the 0.05 level, and * represents the rejection of the null hypothesis at the 0.1 level.

## Appendix A

**Table A1:** The determined hyper-parameters of the experimental models.

Model	Component	Hyper-Parameters	Testing Cases			
			Exit Passenger Flow of Q.R. Sta.	Entrance Passenger Flow of Q.R. Sta.	Exit Passenger Flow of J. Sta.	Entrance Passenger Flow of J. Sta.
ARIMA	-	*p*, *d*, *q*	10,0,3	8,0,7	6,0,10	6,0,9
ANN	-	*H*	24	24	26	34
LSTM	-	*H*	40	34	28	22
ELM	-	*H*	50	50	42	42
AWELM	-	*H, T*	50,13	50,5	50,8	42,16
SSA-AWELM	trend	*H, T*	24,15	22,20	22,13	24,11
	periodicity	*H, T*	50,19	50,15	46,15	48,11
	remainder	*H, T*	46,17	44,9	40,8	48,10

*H* represents the neuron number of the hidden layer, and *T* represents the number of base learners.

**Table A2:** The training time of the experimental models.

Models	Training Time (Seconds)			
	Exit Passenger Flow of Q.R. Sta.	Entrance Passenger Flow of Q.R. Sta.	Exit Passenger Flow of J. Sta.	Entrance Passenger Flow of J. Sta.
**ARIMA**	~10.1	~8.9	~12.8	~11.0
**ANN**	~2.9	~1.6	~3.6	~2.4
**LSTM**	~54.0	~51.8	~51.0	~55.0
**ELM**	<1	<1	<1	<1
**AWELM**	~4.6	~1.7	~2.9	~4.1
**SSA-AWELM**	~9.6	~8.8	~8.2	~7.8

The experiment is conducted in the environment: programming language: *Python*; Main Packages: *Statsmodels*, *Scikit-learn*, *Keras*, and *TensorFlow*; OS: Windows 10 (64bit); RAM: 8G; CPU: Intel Core i5-8300H 2.30 GHz; and GPU: NVIDIA GeForce GTX 1650Ti.

## References

[1] Gallo M., De Luca G., D’Acierno L., Botte M. (2019). Artificial neural networks for forecasting passenger flows on metro lines. Sensors.

[2] Chen Q., Wen D., Li X., Chen D., Lv H., Zhang J., Gao P. (2019). Empirical mode decomposition based long short-term memory neural network forecasting model for the short-term metro passenger flow. PLoS ONE.

[3] Lin P., Weng J., Fu Y., Alivanistos D., Yin B. (2020). Study on the topology and dynamics of the rail transit network based on automatic fare collection data. Phys. A Stat. Mech. Appl..

[4] Zhang J., Wang S., Zhang Z., Zou K., Shu Z. (2016). Characteristics on hub networks of urban rail transit networks. Phys. A Stat. Mech. Appl..

[5] Liu Z.Q., Song R. (2010). Reliability analysis of Guangzhou rail transit with complex network theory. J. Transp. Syst. Eng. Inf. Technol..

[6] Du Z., Tang J., Qi Y., Wang Y., Han C., Yang Y. (2020). Identifying critical nodes in metro network considering topological potential: A case study in Shenzhen city—China. Phys. A Stat. Mech. Appl..

[7] Tang L., Zhao Y., Cabrera J., Ma J., Tsui K.L. (2019). Forecasting Short-Term Passenger Flow: An Empirical Study on Shenzhen Metro. IEEE Trans. Intell. Transp. Syst..

[8] Danfeng Y., Jing W. (2019). Subway Passenger Flow Forecasting with Multi-Station and External Factors. IEEE Access.

[9] Ding X., Liu Z., Xu H. (2019). The passenger flow status identification based on image and WiFi detection for urban rail transit stations. J. Vis. Commun. Image Represent..

[10] Liu S., Yao E. (2017). Holiday passenger flow forecasting based on the modified least-square support vector machine for the metro system. J. Transp. Eng..

[11] Jiao P., Li R., Sun T., Hou Z., Ibrahim A. (2016). Three Revised Kalman Filtering Models for Short-Term Rail Transit Passenger Flow Prediction. Math. Probl. Eng..

[12] Liu Y., Liu Z., Jia R., Deep P.F. (2019). A deep learning based architecture for metro passenger flow prediction. Transp. Res. Part C Emerg. Technol..

[13] Fu X., Gu Y. (2018). Impact of a New Metro Line: Analysis of Metro Passenger Flow and Travel Time Based on Smart Card Data. J. Adv. Transp..

[14] Tavassoli A., Mesbah M., Shobeirinejad A. (2018). Modelling passenger waiting time using large-scale automatic fare collection data: An Australian case study. Transp. Res. Part F Traffic Psychol. Behav..

[15] Xu X., Xie L., Li H., Qin L. (2018). Learning the route choice behavior of subway passengers from AFC data. Expert Syst. Appl..

[16] Hao S., Lee D.H., Zhao D. (2019). Sequence to sequence learning with attention mechanism for short-term passenger flow prediction in large-scale metro system. Transp. Res. Part C Emerg. Technol..

[17] Lee S., Fambro D.B. (1999). Application of subset autoregressive integrated moving average model for short-term freeway traffic volume forecasting. Transp. Res. Rec..

[18] Milenković M., Švadlenka L., Melichar V., Bojović N., Avramović Z. (2018). SARIMA modelling approach for railway passenger flow forecasting. Transport.

[19] Wang Y.H., Jin J., Li M. (2013). Forecasting the section passenger flow of the subway based on exponential smoothing. Appl. Mech. Mat..

[20] Yu B., Song X., Guan F., Yang Z., Yao B. (2016). K-Nearest Neighbor Model for Multiple-Time-Step Prediction of Short-Term Traffic Condition. J. Transp. Eng..

[21] Cai P., Wang Y., Lu G., Chen P., Ding C., Sun J. (2016). A spatiotemporal correlative k-nearest neighbor model for short-term traffic multistep forecasting. Transp. Res. Part C. Emerg. Technol..

[22] Tsai T.H., Lee C.K., Wei C.H. (2009). Neural network based temporal feature models for short-term railway passenger demand forecasting. Expert Syst. Appl..

[23] Zhang Y., Zhang Y., Haghani A. (2014). A hybrid short-term traffic flow forecasting method based on spectral analysis and statistical volatility model. Transp. Res. Part C Emerg. Technol..

[24] Zeng D., Xu J., Gu J., Liu L., Xu G. Short term traffic flow prediction using hybrid ARIMA and ANN models. Proceedings of the 2008 Workshop on Power Electronics and Intelligent Transportation System (PEITS 2008).

[25] Sun Y., Leng B., Guan W. (2015). A novel wavelet-SVM short-time passenger flow prediction in Beijing subway system. Neurocomputing.

[26] Wei Y., Chen M.C. (2012). Forecasting the short-term metro passenger flow with empirical mode decomposition and neural networks. Transp. Res. Part C Emerg. Technol..

[27] Yang D., Chen K., Yang M., Zhao X. (2019). Urban rail transit passenger flow forecast based on LSTM with enhanced long-term features. IET Intell. Transp. Syst..

[28] Bai Y., Sun Z., Zeng B., Deng J., Li C. (2017). A multi-pattern deep fusion model for short-term bus passenger flow forecasting. Appl. Soft Comput. J..

[29] Liu L., Chen R.C. (2017). A novel passenger flow prediction model using deep learning methods. Transp. Res. Part C Emerg. Technol..

[30] Ma X., Dai Z., He Z., Ma J., Wang Y., Wang Y. (2017). Learning traffic as images: A deep convolutional neural network for large-scale transportation network speed prediction. Sensors.

[31] Yang C., Guo Z., Xian L. (2019). Time series data prediction based on sequence to sequence model. IOP Conf. Ser. Mat. Sci. Eng..

[32] Li W., Wang J., Fan R., Zhang Y., Guo Q., Siddique C., Ban X. (2020). Short-term traffic state prediction from latent structures: Accuracy vs. efficiency. Transp. Res. Part C Emerg. Technol..

[33] Liu R., Wang Y., Zhou H., Qian Z. (2019). Short-Term Passenger Flow Prediction Based on Wavelet Transform and Kernel Extreme Learning Machine. IEEE Access.

[34] Chen M.C., Wei Y. (2011). Exploring time variants for short-term passenger flow. J. Trans. Geogr..

[35] Qin L., Li W., Li S. (2019). Effective passenger flow forecasting using STL and ESN based on two improvement strategies. Neurocomputing.

[36] Chen D., Zhang J., Jiang S. (2020). Forecasting the Short-Term Metro Ridership with Seasonal and Trend Decomposition Using Loess and LSTM Neural Networks. IEEE Access.

[37] Mao X., Shang P. (2019). Multivariate singular spectrum analysis for traffic time series. Phys. A Stat. Mech. Appl..

[38] Shang Q., Lin C., Yang Z., Bing Q., Zhou X. (2016). A hybrid short-term traffic flow prediction model based on singular spectrum analysis and kernel extreme learning machine. PLoS ONE.

[39] Guo F., Krishnan R., Polak J. (2013). A computationally efficient two-stage method for short-term traffic prediction on urban roads. Transp. Plan. Technol..

[40] Qiu H., Zhang N., Xu W., He T. (2014). Research of Architecture on Rail Transit’s AFC System. Urb. Rapid Rail Transit.

[41] Taieb S.B. (2012). and Hyndman, R.J. Recursive and Direct Multi-Step Forecasting: The Best of Both Worlds.

[42] Bontempi G., Ben Taieb S., Le Borgne Y.A. (2013). Machine learning strategies for time series forecasting. Lecture Notes in Business Information Processing, LNBIP.

[43] Golyandina N., Nekrutkin V.V., Zhigljavsky A.A. (2001). Analysis of Time Series Structure: SSA and Related Techniques.

[44] Freund Y., Schapire R.E. Experiments with a New Boosting Algorithm. Proceedings of the 13th International Conference on Machine Learning.

[45] Drucker H. Improving regressors using boosting techniques. Proceedings of the 14th International Conference on Machine Learning.

[46] Solomatine D.P., Shrestha D.L. AdaBoost.RT: A boosting algorithm for regression problems. Proceedings of the 2004 IEEE International Conference on Neural Networks.

[47] Shrestha D.L., Solomatine D.P. (2006). Experiments with AdaBoost.RT, an improved boosting scheme for regression. Neural Comput..

[48] Huang G.B., Zhu Q.Y., Siew C.K. Extreme learning machine: A new learning scheme of feedforward neural networks. Proceedings of the 2004 IEEE International Conference on Neural Networks.

[49] Tianchi A. The AI Challenge of Urban Computing. https://tianchi.aliyun.com/competition/entrance/231712/information.

[50] Sun Y., Zhang G., Yin H. (2014). Passenger flow prediction of subway transfer stations based on nonparametric regression model. Discret. Dyn. Nat. Soc..

[51] Harvey A.C. (1990). Forecasting, Structural Time Series Models and the Kalman Filter.

[52] Diebold F.X. (2013). Comparing Predictive Accuracy, Twenty Years Later: A Personal Perspective on the Use and Abuse of Diebold-Mariano Tests. SSRN Electr. J..

[53] Zhang X., Wang J., Gao Y. (2019). A hybrid short-term electricity price forecasting framework: Cuckoo search-based feature selection with singular spectrum analysis and SVM. Energy Econ..

